# Extended mesenteric resection reduces the rate of surgical recurrence in Crohn’s disease: a systematic review and meta-analysis

**DOI:** 10.1007/s00384-025-04845-6

**Published:** 2025-02-25

**Authors:** Sascha Vaghiri, Ali Alipouriani, Wolfram Trudo Knoefel, Hermann Kessler, Dimitrios Prassas

**Affiliations:** 1 Department of Surgery (A), Heinrich-Heine-University, Medical Faculty and University Hospital Duesseldorf, Moorenstr. 5, Bldg. 12.46, 40225 Duesseldorf, Germany; 2 Department for Colorectal Surgery, Digestive Disease Institute, Cleveland Clinic, 9500 Euclid Avenue, Cleveland, OH 44195 USA; 3 Department of Surgery, Katholisches Klinikum Essen, Philippusstift, Teaching Hospital of Duisburg-Essen University, Huelsmannstr, 17, 45355 Essen, Germany

**Keywords:** Crohn’s disease, Mesenteric resection, Recurrence rate, Complications

## Abstract

**Purpose:**

Mesenteric resection in Crohn’s disease (CD) is still controversial and under discussion. We performed a meta-analysis to assess recurrence rates and operative-related morbidity based on the extent of mesenteric resection.

**Methods:**

A comprehensive literature research was conducted until November 2024 using PubMed (Medline), the Cochrane Central trials register, and Google Scholar databases. Studies before the biological era or with Kono-S anastomosis were excluded. Data from comparative studies with reported patient characteristics and outcome results of extended and limited mesenteric resections were extracted and subsequently entered into a pairwise meta-analysis model. Odds ratios (ORs) for dichotomous variables and standardized mean differences (SMDs) for continuous outcomes with 95% confidence intervals (CIs) were calculated. The risk of bias was rated according to ROBINS-I and Rob2 criteria, respectively.

**Results:**

Four non-randomized studies and one randomized trial with a total of 4358 patients (extended mesenteric resection: *n* = 993 versus mesenteric preservation: *n* = 3365) met eligibility criteria and were included. Extended mesenteric resection was significantly associated with reduced surgical recurrence rates compared to mesenteric preservation (OR = 4.94; 95% CI [2.22–10.97]; *p* < 0.001, *I*^2^ = 0%). In terms of endoscopic recurrence, postoperative morbidity, and hospital stay, no significant differences between both groups were noted within the short follow-up period.

**Conclusion:**

Extended mesenteric resection demonstrated a lower surgical recurrence rate in Crohn’s disease, while morbidity rates were comparable to the mesenteric sparing approach, whether extended mesenteric excision should be recommended requires further high-quality randomized trials with long-term follow-up data.

**Supplementary Information:**

The online version contains supplementary material available at 10.1007/s00384-025-04845-6.

## Introduction

Over the course of the last three decades, major breakthroughs have been made, unlocking novel therapeutic strategies for patients with Crohn’s disease (CD). Despite of this, the majority will have to undergo one or more surgeries over their lifetime [1, 2]. As a result, this raises the question of the appropriateness of contemporary surgical methods which, more or less, have not evolved at the same pace. Two distinct stages of the surgical approach can be individually investigated and modified in order to pursue a better outcome, with the first one being the resectional and the second one the reconstructive stage. With regard to the latter, the introduction of the Kono-S anastomotic technique offers a potential option to reduce recurrence rates through the exclusion of the mesentery from the anastomosis and thus the prevention of anastomotic distortion [3, 4]. The resectional stage has traditionally been based on the axiomatic approach of “as much as necessary – as little as possible” referring more to the extent of intestinal length excision rather than the mesenteric radicality. The aspect of the excisional extent of the intestinal mesentery is not new. Based on reports that demonstrate the mesentery of the inflamed bowel as a discrete functional entity that has been associated with disease activity [5], this organ has been targeted as its regulator [6, 7].

With the present work, we sought to meta-analyze all contemporary existing studies comparing limited to extended mesenteric excision of the bowel with regard to its efficiency and safety in patients with CD requiring surgical resection.

## Materials and methods

This meta-analysis was conducted in accordance with the current PRISMA (Preferred Reporting Items for Systematic Reviews and Meta-Analyses) statement and the latest version of the Cochrane Handbook for Systematic Reviews of Interventions, to ensure transparency and adequate study evidence [8, 9].

### Literature search

Two authors (S.V. and A.A.) independently performed a comprehensive research for relevant literature in the PubMed (Medline), the Cochrane Central trials register, and Google Scholar databases until November 9, 2024, through screening of article titles and abstracts. No language restriction was applied. The following medical subject headings were combined with the Boolean operators AND or OR: [(mesenter* resection) AND Crohn’s]. After full-text review, the reference lists of all selected and eligible articles were comprehensively checked for further potentially relevant citations. Disagreements were resolved by discussion and consensus or consultation of a third author (D.P.) if necessary.

### Inclusion and exclusion criteria

All comparative studies such as randomized controlled trials (RCTs), and prospective or retrospective cohort studies reporting clinical outcomes of extended mesenteric resection as the intervention of interest (i.e. mesofascial separation and vascular mesenteric pedicle division at various levels) versus mesenteric preservation (mesenteric resection in bowel proximity [10], comparator) during segmental bowel resection for Crohn’s disease were included. The exclusion criteria were as follows: case/technical reports, editorials or narrative reviews, non-peer-reviewed articles, studies from the pre-biological era, studies comparing Kono-S anastomosis with conventional techniques, and studies that did not report specific outcomes of interest. For data integrity, the reported outcomes of interest in each included study should enable the calculation of odds ratios (ORs) with 95% confidence intervals (CIs). Only studies with adult participants were considered. Discrepancies in study selection were resolved either by consensus or by consultation with an independent third author (D.P.).

### Data extraction and outcomes of interest

Two authors (S.V. and A.A.) independently collected all available and relevant data from studies meeting the inclusion criteria. The extracted data contain the following information: (a) study characteristics (authors, year and country of publication, enrollment period, study design and protocol, number of included patients), (b) patients baseline demographics, comorbidities, disease characteristics and medication, (c) surgical technique and intraoperative details, and (d) postoperative short- and long-term outcomes. If the authors were unable to achieve consistent results during data extraction, an independent third reviewer (D.P.) was consulted for advice. The primary endpoint was the rate of surgical recurrence (defined as the need of reoperation for recurrent CD). The secondary outcomes included endoscopic recurrence (defined as Rutgeerts score from i2 to i4 [11], or modified Rutgeerts’ score of i2b or higher [12] within 6–12 months after index surgery), duration of surgery, protective ostomy, anastomotic leak, transfusion, surgical site infection (SSI), overall morbidity, major complications (according to Clavien-Dino and ACS-NSQIP, respectively [13, 14]), mortality, and length of hospital stay.

### Quality and certainty assessment

The risk of bias for the included non-randomized studies was assessed using the ROBINS-I criteria as recommend in the Cochrane Handbook for Systematic Reviews of Interventions [15]. This tool categorizes the risk of bias in non-randomized studies from low to critical based on seven potential bias domains. In parallel, the risk of bias of in one randomized study was evaluated using the RoB 2 criteria, which categorizes randomized trials into low to high risk of bias based on signaling questions derived from five potential bias domains [16]. The revised AMSTAR 2 instrument was used to critically appraise this meta-analysis [17]. The level of evidence for the significant outcomes was classified into four categories (high, moderate, low, and very low) according to GRADE (The Grading of Recommendations, Assessment, Development, and Evaluation) [18]. Study quality and certainty judgement was performed independently by two authors (S.V and A.A.). Disagreements during this step were discussed and resolved by consensus or reassessment by a third author (D.P.).

### Statistical analyses

Data of interest was analyzed with pairwise meta-analyses. For each primary and secondary outcome, summary treatment effect estimates with 95% confidence intervals (CIs) were calculated. For dichotomous endpoints, the odds ratio (OR) was chosen as the effect measure. Standardized mean differences (SMD) were calculated to analyze continuous outcomes. For continuous variables, the available data on medians and IQRs (interquartile range) have been converted into means and standard deviations applying the methods proposed by Luo et al. and Wan et al. [19, 20]. Using the Cochrane *Q* test (chi-squared test; chi^2^) and the measurement of inconsistency (*I*^2^), the degree of heterogeneity among the included studies was interpreted as follows: 0–40% low heterogeneity and may not be important, 30–60% moderate heterogeneity, 50–90% substantial heterogeneity, > 75% considerable heterogeneity [8]. When heterogeneity was low or moderate (*I*^2^ < 50%), summary estimates were calculated using a fixed-effects method. Where appropriate, subgroup analysis was conducted to ensure the robustness of the overall heterogeneity in the results. Leave-one-out analysis investigated the effect of each study on the pooled outcomes. Statistical significance was defined as *p* values < 0.05 of pooled data. Statistical analysis was performed using RevMan software (version 5.3; Copenhagen: The Nordic Cochrane Centre, The Cochrane Collaboration, 2014).

## Results

### Study and patient characteristics

**Fig. 1:**
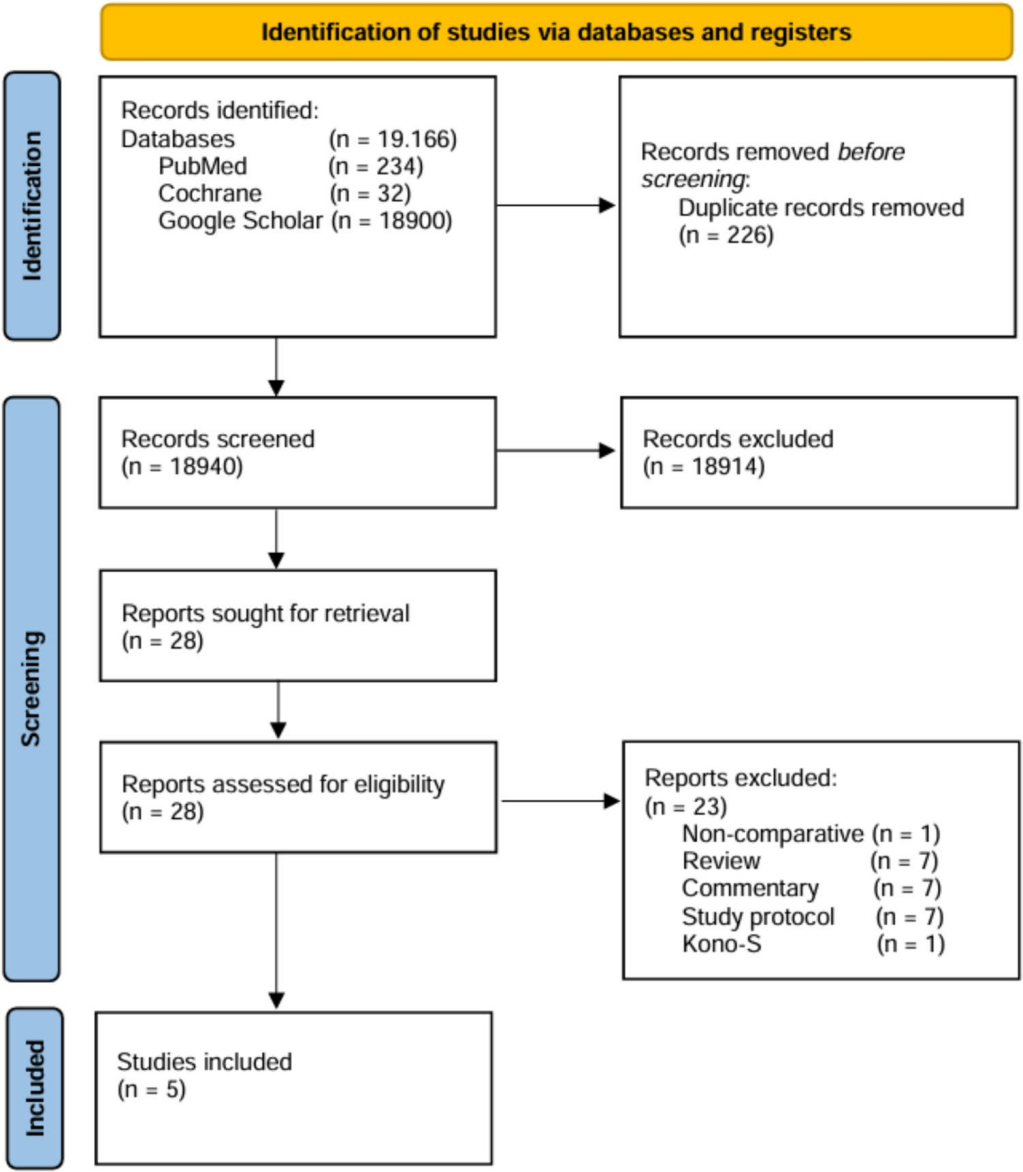
PRISMA flowchart of study identification and selection

The initial literature research resulted in 19,166 studies for inclusion. After removing duplicates and non-contributory studies, 28 full-text manuscripts were screened, 23 of which were excluded taking into account the defined inclusion and exclusion criteria. Finally, five eligible studies involving 4550 patients remained that were subsequently included in the qualitative and quantitative meta-analysis [21–25] (Fig. 1 PRISMA Flowchart). In four non-randomized studies [21–24] and one randomized trial [25], a total of 4358 patients were analyzed (extended mesenteric resection: *n* = 993 versus mesenteric preservation: *n* = 3365). The male to female ratio was 2072:2286. The study enrollment period was from January 2004 to April 2023. Three studies originated from European countries [21, 23, 25], while one study was conducted in Canada [24] and one in China [22], respectively. The study of van der Does de Willeboise et al. [25] was the only included multicenter RCT from the Netherlands with 133 patients. Abdulkarim et al. [24] performed their study as a retrospective ACS database query including 3709 patients. Two studies were conducted as single-center studies [21, 22] and one as a bi-center study [23]. Patients exclusively undergoing ileocolic resection were included in three studies accounting for 12% of all performed procedures [21, 23, 25]. In contrast, two studies included patients with a various extent and location of colectomy (right colectomy 60.6%, transverse colectomy 0.2%, left colectomy 0.8%, total colectomy 0.5%, proctectomy 0.1%, and other non-defined segmental resections 25.9%) [22, 24]. The application of biologicals at index surgery was mentioned in four studies with a total of 136 patients which corresponds to 21% of all patients in these studies [21–23, 25]. Noteworthy, in two studies, follow-up ranged from 30 days to 6 months [24, 25], in contrast to long-term outcome data provided in three other studies [21–23]. A full and detailed study and patient summary is presented in Tables 1 and 2.

**Table 1:** Study characteristics and protocols

Author	Year	Origin	Recruitment period	Study design	Total sample size	Inclusion criteria	Type of procedure (groups)	Type of access/surgeons involved	Follow-up period (months) mean/SD	Endpoint(s)
Coffey et al. [21]	2018	Ireland	Jan 2004–Apr 2010	Single center, retrospective	64	Ileocolic resection for a Crohn’s related ileocolic disease	Group A: conventional ileocolic resection Group B: ileocolic resection with mesenteric excision	Open and MIS/multiple	Group A: 69.9 ± 48.47 Group B: 51.7 ± 20.98	Surgical recurrence
Zhu et al. [22]	2021	China	Jan 2000–Dec 2018	Single-center, prospective	126	Crohn’s colitis with colorectal resection	LME: mesentery sparing colorectal resection EME: mesentery mobilization and division 1-cm distant from the origin of the major arterial trunks	NA	LME: 45.12 6 ± 25.45 EME: 47.50 ± 623.67	Early postoperative short-term outcome and surgical recurrence
Mineccia et al. [23]	2022	Italy	Jan 2009–Dec 2019	Bi-center, retrospective (Remedy study)	326	Crohn’s disease localized to the terminal ileum	Group A: mesentery resection Group B: mesentery preservation	Open and MIS/multiple	Median 4.7 ± 3 (years)	90-day complications, endoscopic FU, long-term surgical recurrence
Abdulkarim et al. [24]	2023	Canada	2014–2019	ACS-NSQIP database, retrospective	3709	Patients with CD undergoing segmental colectomy	Extended ME: lymph node harvest ≥ 12 Limited ME: < 12 lymph node	Open and MIS/multiple	30 days	30-day NSQIP major morbidity, abdominal complications, perioperative bleeding
van der Does de Willebois et al. [25]	2024	Netherlands	Feb 2020–Apr 2023	Multicenter, RCT (SPICY trial -NCT 04538638)	139	Aged ≥ 16 and Crohn’s disease (L1 or L3 disease)	Extended mesenteric resection (intervention) or conventional mesenteric sparing resection (control)	MIS/multiple	6 months	Endoscopic recurrence 6 months after surgery

*CD*, Crohn’s disease; *FU*, follow-up; *EME*, extended mesenteric excision; *LME*, limited mesenteric excision; *MIS*, minimally invasive surgery; *NA*, not available; *NSQIP*, National Surgical Quality Improvement Program; *RCT*, randomized controlled trial; *SD*, standard deviation

**Table 2:** Patient-and disease characteristics

Author	Groups	No. of patients	Age at surgery (years) mean/SD	Gender (M/F)	Disease duration (months) mean /SD	Biologicals at index surgery	Smoking	Disease classification				Site of surgery
								Age	Location		Phenotype	
Coffey et al. [21]	LME	30	37.7 ± 13.67	14/16	75.0 ± 117.42	5	20	A1 < 40 23* A2 ≥ 40 6		L1 23 L2 2 L3 4 L4 0	B1 16 B2 6 B3 8	Ileocolic resection 30
	EME	34	35.9 ± 11.87	14/20	70.7 ± 78.83	15	20	A1 < 40 26 A2 ≥ 40 6		L1 26 L2 0 L3 6 L4 2	B1 8 B2 14 B3 12	Ileocolic resection 34
Zhu et al. [22]	LME	60	31.15 ± 10.36	47/13	51.19 ± 49.36	4	9	A1 ≤ 16 7† A2 17–40 44 A3 > 40 12		L1 0 L2 32 L3 28 L4 1	B1 0 B2 20 B3 40	Right colectomy 26 Transverse colectomy 4 Left colectomy 21 Proctectomy 2 Total colectomy 7
	EME	66	30.42 ± 10.15	42/24	61.61 ± 52.19	4	5	A1 ≤ 16 4 A2 17–40 50 A3 > 40 12		L1 0 L2 41 L3 25 L4 4	B1 3 B2 39 B3 24	Right colectomy 32 Transverse colectomy 3 Left colectomy 14 Proctectomy 1 Total colectomy 16
Mineccia et al. [23]	LME	122	40.7 ± 16	70/52	7.7 ± 8.6 (years median)	16	35	A1 ≤ 16 15† A2 17–40 68 A3 > 40 39		NA	B1 0 B2 40 B3 82	Ileocolic resection 122
	EME	204	40.5 ± 14.7	121/83	7.5 ± 8.3 (years median)	32	75	A1 ≤ 16 12 A2 17–40 140 A3 > 40 52		NA	B1 0 B2 67 B3 137	Ileocolic resection 204
Abdulkarim et al. [24]	LME	3087	42.3 ± 16.76	1408/1679	NA	NA	697	NA		NA	NA	Right colectomy 2165 Other segmental colectomy 922
	EME	622	41.0 ± 15.78	299/323	NA	NA	123	NA		NA	NA	Right colectomy 416 Other segmental colectomy 206
van der Does de Willeboise et al. [25]	LME	66	36.29 ± 18.18	28/38	59.47 ± 85.64	25	25	A1 ≤ 16 8† A2 17–40 40 A3 > 40 18		L1 40 L3 26	B1 15 B2 29 B3 22	Ileocolic resection 66
	EME	67	40.58 ± 23.48	29/38	58.99 ± 61.36	35	30	A1 ≤ 16 4 A2 17–40 38 A3 > 40 25		L1 44 L3 23	B1 22 B2 28 B3 17	Ileocolic resection 67

*EME*, extended mesenteric resection; *LME*, limited mesenteric resection; *SD*, standard deviation; *NA*, not available; *ME*, mesenteric excision

^*^Vienna Classification

^†^Montreal Classification

### Study quality and risk of bias

**Fig. 2:**
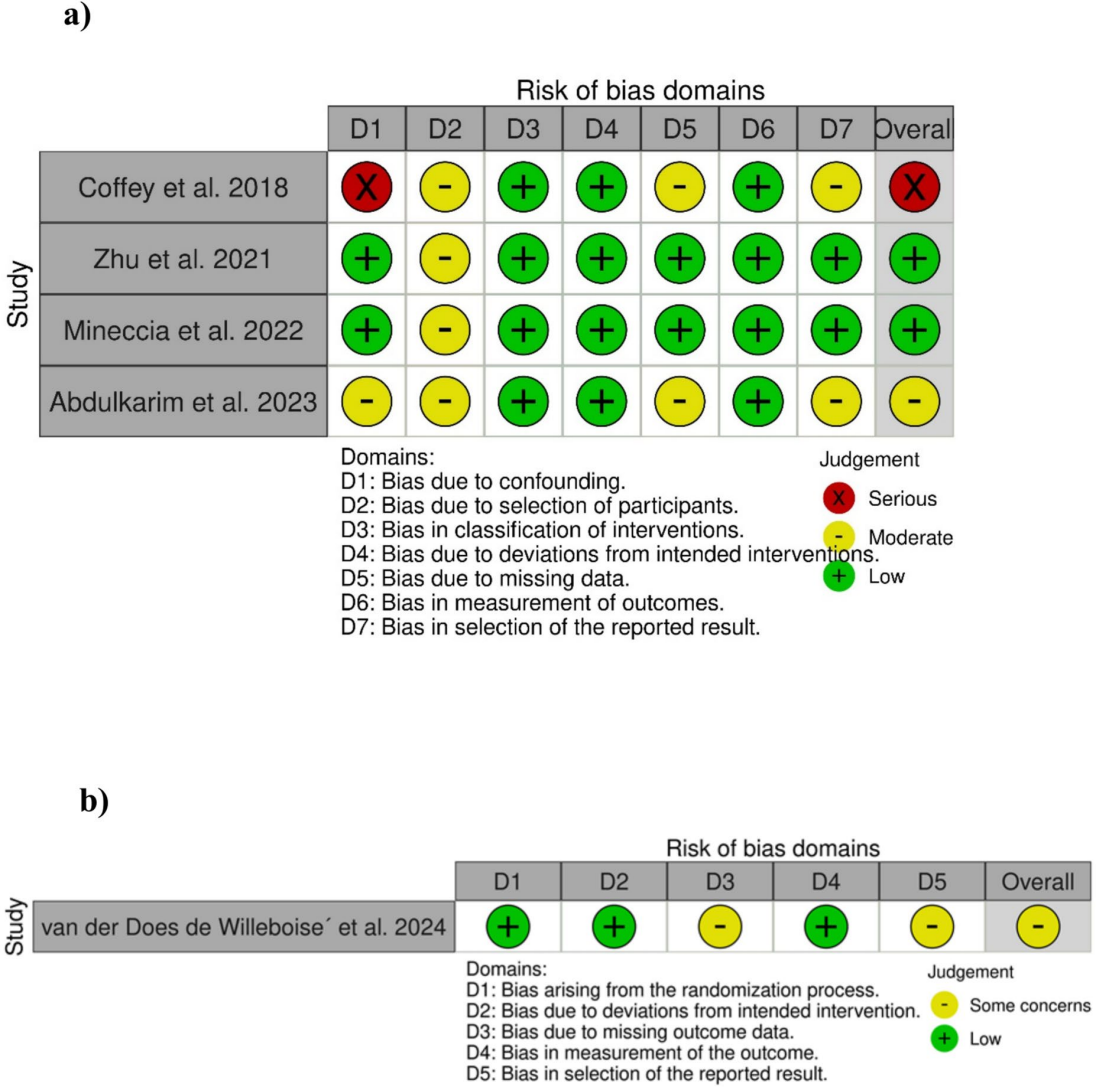
Risk of bias summary according to **a** ROBINS-I, **b** RoB2

The overall risk of bias of the four included non- randomized studies was rated from serious to low based on the proposed seven bias domains in the ROBINS-I tool. The risk of bias in the domain of confounding was serious in one study [21], moderate in one study [24] and low in two studies [22, 23]. The risk of bias in the domain selection of study participants was judged as moderate in all studies [21–24]. The risk of bias arising from classification of interventions, deviations from intended interventions, and measurement of outcomes was rated low in all non-randomized studies [21–24]. Two studies were observed to have moderate risk of bias in the domains missing outcomes and selection of reported results [21, 24]. Furthermore, the only randomized study was analyzed as having some risk of bias concerns especially in the categories missing outcome data and selection of the reported results, respectively [25]. The methodological quality of the pooled evidence in this meta-analysis was determined as “high” using the AMSTAR 2 quality assessment tool. The risk of bias evaluation is demonstrated in Fig. 2.

### Primary outcome

#### Surgical recurrence

**Fig. 3:**
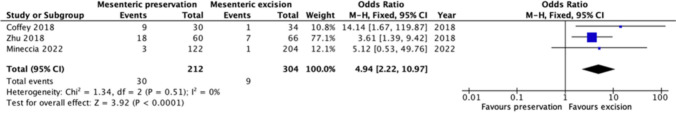
Forrest plot for primary outcome extended mesenteric resection versus control: surgical recurrence

Surgical recurrence as the primary outcome was reported in three studies with a total of 416 patients [21–23]. Meta-analysis of the pooled data revealed a significantly lower rate of surgical recurrence associated with extended mesenteric resection compared to the control group (OR = 4.94; 95%CI [2.22–10.97]; *p* < 0.0001). The rate of heterogeneity was notably low (*I*^2^ = 0%, chi^2^ test: *p* = 0.51) (Fig. 3). The certainty of evidence was judged as high based on GRADE criteria (Table suppl. 1).

#### Secondary outcomes

Non-significant differences between extended mesenteric resection and mesenteric preservation were observed for all secondary outcomes of interest, especially overall and severe postoperative morbidity was not increased with extended mesenteric resection. The results are presented in Table 3.

**Table 3:** Non-significant secondary outcomes

		No. of included patients				Heterogeneity level	
Outcomes	No. of included studies	Mesenteric preservation	Mesenteric excision	SMD/OR [95% CI]	*P*-value	*I*^2^ (%)	*P*-value
Endoscopic recurrence	2 [23, 25]	187	270	1.07 [0.73–1.56]	0.72	0	0.89
Surgery duration (min)	3 [23–25]	3274	892	− 0.04 [− 0.12–0.03]	0.26	0	0.48
Protective ostomy	2 [22, 24]	3147	688	1.10 [0.80–1.52]	0.56	0	0.44
Anastomotic leak	3 [22, 24, 25]	2980	721	1.06 [0.35–3.24]	0.91	51	0.13
Transfusion	2 [22, 24]	3147	688	0.86 [0.63–1.18]	0.36	0	0.77
Surgical site infection	2 [22, 24]	3147	688	1.00 [0.77–1.31]	0.99	0	0.60
Overall morbidity	3 [22–24]	3269	892	1.13 [0.94–1.37]	0.21	0	0.71
Major morbidity	3 [23–25]	3274	892	1.01 [0.81–1.26]	0.90	0	0.48
Mortality	4 [22–25]	3334	958	1.96 [0.27–14.52]	0.51	0	0.89
Length of hospital stay (days)	4 [22–25]	3334	958	0.02 [− 0.06–0.09]	0.69	28	0.24

*CI*, confidence interval; *OR*, odds ratio; *SMD*, standardized mean difference

## Discussion

Extended mesenteric excision is not a novel concept [26], but is increasingly utilized, in conjunction with modern medical treatment regimens and alternative anastomotic approaches, as an efficient amendment to the classic tubular bowel resection. The mesentery-preserving strategy is based on the hypothesis that the biological profile of CD is mostly dependent on gut microbiome and the intestinal barrier. Coffey et al. [27] revitalized the interest towards the mesentery as a distinct anatomic and pathophysiologic entity by underlining its role in metabolism and as mediator of immune response. Increasing evidence shows that structural abnormalities of the mesentery are closely linked to disease progression and recurrence, rendering it a key mediator of inflammatory activity due to interaction between adipocytes, neuropeptides and lymphatic and vascular endothelia, causing remodeling of the involved adipose tissue [28–31]. Again, Coffey et al. [7, 27] were the first to revive this concept and implement it into surgical practice by conducting the first contemporary comparative study of tubular versus extended resections for CD, with more studies following. Here, it should be highlighted that the specimen length was mentioned in only three included studies [21, 23, 25] and two authors provided data on the resection status [22, 25]. In two studies [21, 23], there was a clear trend towards a longer specimen length in the limited mesenteric approach, while in the study by van der Does de Willeboise et al. [25] only the ileal specimen was longer in the mesenteric sparing group. On the other hand, in the study by Zhu et al. [22], the CD affected resection margins did not differ significantly between the two groups with limited and extended resection, while van der Does de Willeboise et al. [25] reported a higher inflammatory involvement of the distal colonic margin in the group with limited resection as opposed to extended mesenteric excision (18% versus 5%, *p* = 0.023). Interestingly, recurrence rates do not appear to correlate with margin status and specimen length based on the data of the included studies. This in turn is a contrast to a recently published meta-analysis, demonstrating inflammatory margins to be associated with postoperative recurrence after ileocecal resection in CD [32]. At this point, it must be noted that the term radical excision does not always imply the exact same operative technique. Zhu et al. [22] and Mineccia et al. [23] define it as full mesenteric mobilization with division in close proximity to the major arterial trunks, similar to oncologic colorectal resections, whereas Coffey et al. [21] describe his technique as division of the mesentery as close to the mesenteric root as deemed safe. On the other hand, the mesenteric resections in the SPICY study [25] were performed following the lower border of the ileocolic artery, preserving its vascular trunk. The lack of predefined mesenteric borders of the resection is most prominent in the study of Abdulkarim et al. [24], in which lymph node harvest alone was used as a surrogate for radicality. This is the first meta-analysis of studies comparing the above-mentioned procedures. Upon extensive literature research, six comparative contemporary studies and one trial from 1989 [26] were identified. In order to eliminate the potential heterogeneity generated by the synergistic effects of concomitant medications of different classes on treatment response, we opted for exclusion of trials published in the pre-biologic era [33]. This exclusion criterion becomes more important when one considers that in Coffey’s study, 44.1% of patients in the mesenteric excision group (recurrence rate 3%) had biologic therapy at index surgery compared to only 16.7% in the non-excision group which is further related to the different time periods of surgery in both groups [34]. The Kono-S anastomosis has been shown to have a positive effect on reducing disease recurrence [35, 36], so we excluded one study comparing the Kono-S, extended mesenteric resection and the combination of Kono-S and extensive mesenteric resection techniques to ensure homogeneity and comparability [37]. Our meta-analysis demonstrated homogeneous results in support of extended mesenteric resection with regard to clinical recurrence requiring surgery (OR = 4.94; 95% CI [2.22–10.97]; *p* < 0.001, *I*^2^ = 0%). Endoscopic recurrences were reported in just two of the included studies [23, 25]. Despite the higher incidence in the non-excisional subgroup, this effect did not manage to reach statistical significance. One possible explanation of this finding would be the relatively short follow-up period of six months in the SPICY trial [25]. This becomes clear when one considers the recurrence rates of up to 40% in patients with negative initial postoperative surveillance endoscopy, after a median follow-up period of 3.5 years [38]. Furthermore, in light of our results regarding surgical recurrence, the fact that disease progression is linear suggests that endoscopic recurrence could be higher at longer surveillance. Long-time results of the SPICY trial are yet to be published [39]. Another important clue is the different endoscopic scoring system in the two included studies in which endoscopic recurrence was investigated. While Mineccia et al. [23] used the Rutgeerts score for endoscopic recurrence evaluation, in the study of van der Does de Willebois et al. [25], endoscopic recurrence was judged by the modified Rutgeerts classification. An accurate diagnosis and classification of the endoscopic recurrence is of the utmost importance, as it determines the further disease course, the type of medical treatment, and the quality of life [40]. In this context, a recent meta-analysis of 76 studies showed a wide variation in endoscopic recurrence (5–93%) after surgical resection for CD, depending on the endoscopic scoring system used [41]. Ultimately, the identification of high-risk individuals with regard to recurrence and CD-related morbidity is the fundamental aim of postoperative management. The guidelines published by the American Gastroenterological Association in 2017 have generally served as the guiding beacon for stratifying and accordingly treating patients at risk, and have recently been renewed by the new practice guideline published in 2024 [42]. Studies included in the present meta-analysis demonstrate a rather inhomogeneous pattern with regard to the above matter, reflecting the need for a more standardized algorithmic approach of postoperative medical management. There was no statistically significant difference noted, concerning overall postoperative morbidity within the two study groups. Even after investigating various postoperative complications, still both approaches were found to deliver comparable results. Nevertheless, most of those secondary outcome assumptions derive from pooling of just two studies and should be interpreted cautiously. The presented finding could have a trend-setting impact on the current surgical practice in CD. The latest European Crohn’s and Colitis Organization (ECCO) guidelines state that there is insufficient evidence supporting extended mesenteric excision in ileocecal CD [43]. Recent studies have shown that postoperative disease recurrence is significantly lower depending on the type of anastomotic technique and the extent of resection margin involvement [44, 45], indicating that surgical procedures for CD can be further improved and optimized [46].

Our meta-analysis has some limitations. Firstly, the number of studies that could be included was relatively low. Surgical expertise of the operating surgeons was variable and thus a potential source of bias. The heterogeneity of the present meta-analysis with regard to inclusion of non-exclusively ileocecal resections and the use of different operative techniques may influence comparability of the presented results. Postoperative surveillance strategies, medical treatment regimens that change even within the same study period and follow-up time vary significantly. In addition, positive resection margins should be taken into consideration in future comparative trials as a significant confounding factor for disease recurrence, perhaps by excluding selected cases and/or matching between study arms as appropriate. Only one of the included studies was a randomized controlled trial [25]. Long-time results of this trial, as well as preliminary results of other ongoing RCTs are yet to be published [47–49].

## Conclusion

Extended mesenteric resection was associated with a reduced incidence of postoperative surgical recurrence of Crohn’s disease, without demonstrating any additional morbidity. Due to the lack of high-quality trials, non-standardized operative technique, varying postoperative endoscopic surveillance classification, and heterogeneity of postoperative medical treatment, the results should be interpreted with caution.

## Supplementary Information

Below is the link to the electronic supplementary material.

Supplementary file1 - PRISMA checklist (DOCX 31.4 KB)

Supplementary file2 - The GRADE Certainty assessment for significant outcomes (DOCX 14.3 KB)

## Data Availability

No datasets were generated or analysed during the current study.
