# Supplementary material for: Extended mesenteric resection reduces the rate of surgical recurrence in Crohn’s disease: a systematic review and meta-analysis
Source: Int J Colorectal Dis. 2025 Feb 25;40(1):51. doi: 10.1007/s00384-025-04845-6 (PMC11861228; doi:10.1007/s00384-025-04845-6)
Supplement: Supplementary file 2 — Supplementary file2 - The GRADE Certainty assessment for significant outcomes (DOCX 14.3 KB) [file 384_2025_4845_MOESM2_ESM.docx]

| Table Suppl. 1 The GRADE Certainty assessment for the significant outcomes | | | | | | | | | | |
| --- | --- | --- | --- | --- | --- | --- | --- | --- | --- | --- |
| Outcomes | No. of studies | **No. of included patients** | | OR [95 % CI] | **Quality assessment** | | | | | Quality |
|  |  | Mesenteric preservation | Mesenteric excision |  | Risk of bias^a^ | Inconsistency | Indirectness | Imprecision | Publication bias |  |
| Surgical recurrence | 3 [21,22,23] | 212 | 304 | 4.95 [2.22-10.97] | Not serious | Not serious | No indirectness | No imprecision | NA | High |
| NA: not applicable, OR: odds ratio, SMD: standardized mean difference,  a Risk of bias assessed using the ROBINS-I/Rob2 tools | | | | | | | | | | |
